# Are Infants and Children at Risk of Adverse Health Effects from Dietary Deoxynivalenol Exposure? An Integrative Review

**DOI:** 10.3390/ijerph21060808

**Published:** 2024-06-20

**Authors:** Susan Gonya, Pamela Kallmerten, Pamela Dinapoli

**Affiliations:** Department of Nursing, College of Health and Human Services, University of New Hampshire, Durham, NH 03824, USA

**Keywords:** infants, children, diet, deoxynivalenol, vomitoxin, mycotoxins, fungi, gastrointestinal disorders, immune dysfunction, microbiome

## Abstract

Deoxynivalenol (DON) is a foodborne mycotoxin produced by *Fusarium* molds that commonly infect cereal grains. It is a potent protein synthesis inhibitor that can significantly impact humans’ gastrointestinal, immune, and nervous systems and can alter the microbiome landscape. Low-dose, chronic exposure to DON has been found to stimulate the immune system, inhibit protein synthesis, and cause appetite suppression, potentially leading to growth failure in children. At higher doses, DON has been shown to cause immune suppression, nausea, vomiting, abdominal pain, headache, diarrhea, gastroenteritis, the malabsorption of nutrients, intestinal hemorrhaging, dizziness, and fever. A provisional maximum tolerable daily intake (PMTDI) limit of 1 µg/kg/body weight has been established to protect humans, underscoring the potential health risks associated with DON intake. While the adverse effects of dietary DON exposure have been established, healthcare communities have not adequately investigated or addressed this threat to child health, possibly due to the assumption that current regulatory exposure limits protect the public appropriately. This integrative review investigated whether current dietary DON exposure rates in infants and children regularly exceed PMTDI limits, placing them at risk of negative health effects. On a global scale, the routine contamination of cereal grains, bakery products, pasta, and human milk with DON could lead to intake levels above PMTDI limits. Furthermore, evidence suggests that other food commodities, such as soy, coffee, tea, dried spices, nuts, certain seed oils, animal milk, and various water reservoirs, can be intermittently contaminated, further amplifying the scope of the issue. Better mitigation strategies and global measures are needed to safeguard vulnerable youth from this harmful toxicant.

## 1. Introduction

Humans are continuously exposed to mycotoxins in the natural environment, particularly from foods. While their role in the development of various pediatric diseases is often overlooked, it is important to note that some highly prevalent foodborne mycotoxins pose considerable health risks for vulnerable populations—particularly infants and children, owing to their low body weights relative to the toxin load [1].

Mycotoxins are toxic secondary metabolites produced by filamentous fungi [2]. Hundreds of mycotoxins have been identified since the 1960s, with the most common route of exposure being dietary contact. However, inhalation and contact exposure can also occur in mold-contaminated spaces (e.g., water-damaged homes) and within working environments (e.g., breweries and bakeries) [2,3,4].

*Aspergillus*, *Penicillium*, and *Fusarium* molds are the three most common fungal genera infecting the human food supply [2]. These mold species produce harmful mycotoxins, such as aflatoxins, ochratoxins, trichothecenes (e.g., deoxynivalenol (DON), nivalenol (NIV), and T-2 toxin), and fumonisins, among others [2]. Many of these mycotoxins can adversely alter the structure and functions of various body systems, including the intestines, liver, and renal epithelia, as well as the nervous, reproductive, and immune systems [5,6,7,8]. These effects can affect infants and children more severely than adults owing to developmental issues, such as their immature metabolic, immune, and nervous systems; the reduced ability to metabolize and detoxify toxins; and their smaller body size [1,9].

Among the most common foodborne mycotoxins are the trichothecenes produced by *Fusarium* molds. *Fusarium* molds routinely infect grains and other crops in the field and during storage and produce potent trichothecene mycotoxins, such as DON, NIV, zearalenone (ZEA), HT-2 toxin, T-2 toxin, and diacetoxyscirpenol, among others [10,11,12]. Among the trichothecene mycotoxins, DON is the most prevalent and has been found in the highest concentrations in food [12].

DON is also known as vomitoxin owing to its emetic effects on the gastrointestinal tract [11,13]. Low-dose, chronic exposure to DON has been found to stimulate the immune system, inhibit protein synthesis, and cause appetite suppression, potentially leading to growth failure in children [12,14,15,16,17]. At higher doses, DON has been shown to cause immune suppression, nausea, vomiting, abdominal pain, headache, diarrhea, gastroenteritis, the malabsorption of nutrients, intestinal hemorrhaging, dizziness, and fever, among other adverse sequelae in animals and humans [5,15,18,19,20]. 

Because of DON’s adverse effects on the gastrointestinal tract, immune system regulation, nutrient absorption, and protein synthesis, some scientists have theorized that chronic exposure could contribute to the symptoms experienced by those with inflammatory diseases of the gastrointestinal tract, such as inflammatory bowel disease, celiac disease, and irritable bowel syndrome [5,10,11,13,18]. In a recent study in Shandong province in China, in areas with a high prevalence of DON-contaminated wheat, adverse health effects (e.g., vomiting and diarrhea) were observed in more than 100 in 100,000 residents, and these were increased in wheat-growing areas during heavy precipitation years, with 300/100,000 extra cases [13]. In addition to these adverse sequelae, DON can alter intestinal microbial populations in an antibiotic-like fashion, dramatically affecting resident microbes and the integrity of the intestinal mucus barrier, which enables it to become bloodborne [21,22,23,24]. 

Once bloodborne, it can also cross the blood–brain barrier (BBB), potentially adversely affecting peripheral and central nervous system tissues in infants and children [17,25,26,27]. DON’s detrimental effects on myenteric glial cells and the central nervous system have led some researchers to postulate that it could function as an environmental factor in pediatric nervous system-related diseases, such as autism spectrum disorder (ASD) [17,25,26,27].

These adverse effects can begin to influence the development of children before birth. In a study of the mycotoxin content of amniotic fluid samples (n = 86), DON was found in 27.9% of samples [28]. Maternal DON exposure, in turn, appears to adversely affect fetal development [29]. In a recent prospective cohort study by Tan et al. (2023), mothers in the highest tertile of DON intake were more likely to have infants with lower birth weights and decreased birth lengths that were low for the infant’s gestational age (all P-trends < 0.05) [29].

### Provisional Maximum Tolerable Daily Intake (PMTDI) Levels

At the turn of this century, DON’s adverse effects on humans prompted the establishment of tolerable daily intake (TDI) limits of 1.1 micrograms/kilogram (µg/kg) of human body weight (bw) [30]. The original TDI was derived from data on probabilistic exposure levels with large confidence intervals to protect humans and did not include acetylated derivatives of DON [30]. To stay within the PMTDI recommendations, a maximum concentration limit of DON of no more than 129 µg/kg in individual foods eaten by children was recommended [30]. Since then, additional investigations into DON’s toxic effects have prompted governing agencies to adjust this limit downward. The Joint Food and Agricultural Organization/World Health Organization (WHO) and the Expert Committee on Food Additives updated recommendations in the 2010 Codex Alimentarius, amending the total PMTDI for DON to 1 µg/kg/bw/day and added to these limits its masked acetylated forms (i.e., 3-ADON, 15-ADON, DON-3G, and DON-15G), which can be similarly toxic [31,32]

The Food and Agricultural Organization of the United Nations and the United States (US) Food and Drug Administration (FDA) have also issued guidelines for safety limits for animal feed and human food [31,33]. Table 1 highlights the human exposure limits set in Europe and by the US FDA for DON and other foodborne mycotoxins. Acetylated derivatives are not included in these numbers, although 10–20% of DON is believed to exist in acetylated forms that mask its presence in foods [33,34].

However, these limits were set higher than PMTDI recommendations and might not be strict enough to protect children owing to the excessive contamination rates in the human food supply. Researchers have reported that levels that far exceed the PMTDI might be unavoidable in some populations, and especially in children, owing to their high intake of cereal grains relative to their low body weight, placing them at risk of high-dose chronic exposure and significant harm [31]. To our knowledge, while scientific and agricultural communities are aware of the adverse effects of dietary mycotoxin exposure on the health and well-being of animals and humans, DON has not been recognized by most healthcare communities as a threat to child health. This may stem from the belief that current regulatory exposure limits adequately protect the public.

The primary objectives of this integrative review were to search the literature for evidence of widespread DON contamination in the food supply and to assess whether current global contamination rates place infants and children at risk of ill health effects. To help assess the risks to infant and child health, the following questions were asked: (1) Where might infants and children be exposed to DON in the food supply? (2) Do current contamination rates and dosages exceed safe limits for pediatric DON consumption based on the established PMTDI limits?

## 2. Materials and Methods

An integrative review of the literature from 2000 to 2023 was conducted to review the toxic effects of DON on humans and identify where children are most likely to be exposed to DON in the food supply. The Medline, PubMed, and Biological Science Collection databases were searched using the following keywords or phrases both individually and in varying combinations: deoxynivalenol, DON, trichothecene, mycotoxin, food, grains, corn, wheat, water, tea, coffee, spice, nuts, milk, meat, fusarium, infection, food contamination, and crop contamination.

**Figure 1:**
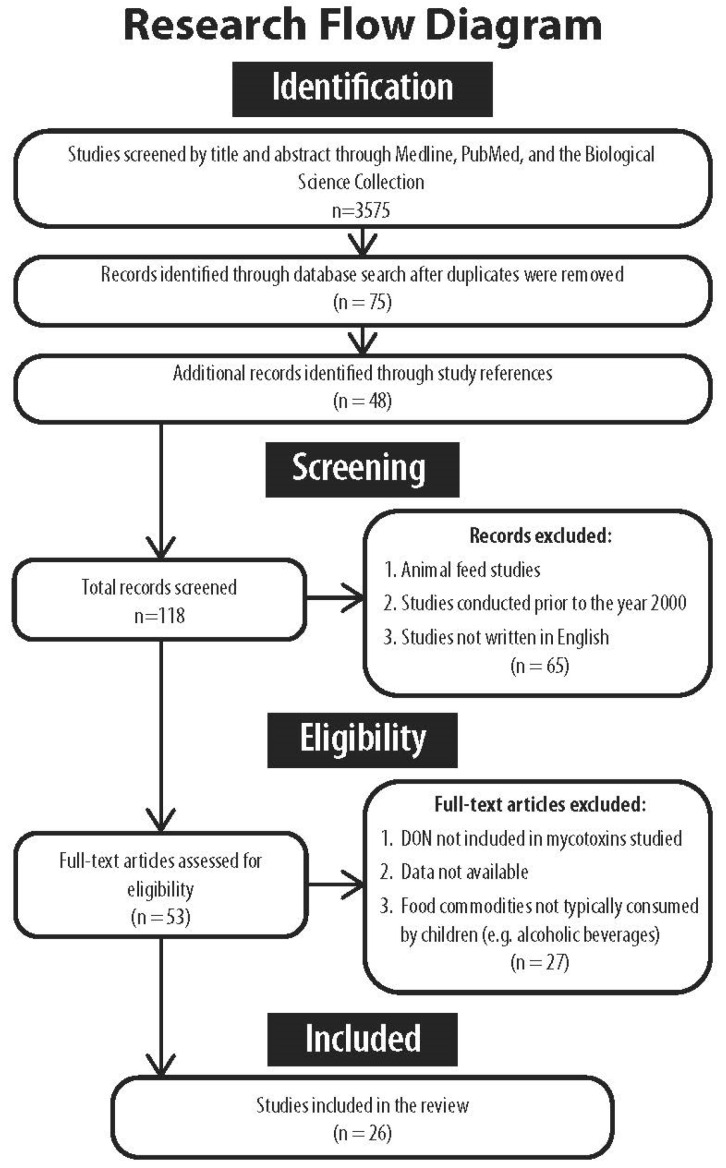
PRISMA diagram.

Occurrences and incidences of DON contamination globally in raw, cooked, baked, processed, and unprocessed foods were sought. In total, 3575 titles and abstracts were initially screened. Studies showing infection occurrence anywhere from farm to storage to table were included. Only those that assessed contaminated foods for human consumption were included. The literature was also assessed for the quality of methods, and only studies that used validated and reliable testing methods (e.g., lateral flow screening or liquid chromatography combined with mass spectrometry–mass spectrometry (LC-MS/MS)), random sampling, and adequate control procedures to limit bias were included. Surveys and analyses of cereals and other commodities intended for animal feed were excluded, as more permissive regulatory guidelines apply to these commodities (e.g., 5000 µg/kg for swine versus 1000 µg/kg for human consumption) [33]. Studies not written in English were also excluded. In total, 118 studies were initially reviewed. The Preferred Reporting Items for Systematic Reviews and Meta-Analysis (PRISMA) diagram in Figure 1 displays the flow of research selection for crop contamination rates. Ultimately, one comprehensive systematic review covering global contamination incidences from 2008 to 2019, and 25 other original studies identifying DON, its acetylated metabolites, and other trichothecene mycotoxins, were reviewed for crop contamination rates. DON occurrence and detected levels were evaluated against the currently established PMTDI intake levels.

The data presented in various studies were heterogeneous regarding the units of measure. All data retrieved from studies were mathematically converted to microgram units for ease of clinical interpretation and comparisons to the PMTDI and current regulatory guidance levels. Only studies that provided descriptive statistics and mean data were included in table format. In addition, when multiple samples were available for a commodity, the data were pooled, and the percentage of positive samples was weighted based on the sample size to calculate the table data. Studies that provided median and range data were summarized narratively.

Based on data retrieved on global exposure levels, a pediatric health risk assessment was conducted by comparing food levels in pediatric portion (e.g., 30 g) sizes to current PMTDI recommendations using an algorithm [38]. The equation was developed by the Italian National Institute of Health, the Department of Innovation in Biology, University of Tuscia, Italy, and an Italian food company to develop processed wheat-based products with child-safe DON levels [38]. This equation provides a method for comparing the mean DON levels found in food with the current PMTDI. The following equation was used to calculate exposure risk in children:

*Exposure* ng/kg/bw/day = *DON Contamination level* (µg/kg) × *Consumption data* (g)/bw (kg).

To use the equation, the DON levels found in food were multiplied by the weight of the food in grams (g) divided by body weight (bw) in kilograms (kg). The total intake of DON in nanograms (ng) per kg/bw/day was then further divided by 1000 to provide the intake levels in µg/kg/bw/day for comparison with the PMTDI limit of 1 µg/kg/bw/day [38]. Infant and child population weights for age were obtained from the Center for Disease Control and Prevention (CDC) growth charts, and the 50th percentile was used for the population risk estimation [39]. 

Microsoft Excel was used to calculate the PMTDI exposure rates for various commodities. This method allowed for easy comparison between the DON exposure levels from foods and the PMTDI limit. All pediatric exposure levels above the PMTDI guidelines were considered a risk to child health.

## 3. Results and Discussion

### 3.1. Survey of DON Contamination Rates and Levels in Global Food Supply

Multiple crops and food commodities were contaminated with DON in the field or during storage. These crops and foods included wheat, corn, barley, rye, oats, millet, triticale, rice, sorghum, soy, coffee, tea, dried spices, nuts, and certain seed oils [12,31,40,41,42,43,44,45]. In addition, DON in its modified forms and other co-contaminant mycotoxins, such as aflatoxin, ochratoxin A, fumonisins, NIV, T-2 toxin, patulin, and some others, were also frequently identified.

Globally, wheat, corn, bakery products, pasta, and mothers’ milk were found to be routinely contaminated with DON. There was sufficient evidence to suggest that other non-grain-based foods, such as soy, coffee, tea, dried spices, nuts, certain seed oils, animal milk, and various water reservoirs, are also intermittently contaminated. Ready-prepared foods, including cereals, pasta/noodles, and bakery products, were routinely contaminated, and rice was intermittently contaminated with DON [31,46]. Furthermore, virtually any food that could have been transported or stored for extended periods without controlled climate conditions related to moisture and temperature appeared vulnerable to mold contamination [36]. For example, DON production in wheat was found to increase by 13–34% after 3 months in storage and by 24–57% at 6 to 12 months, depending on climate conditions [47]. Table 2 shows the results of the synthesized data.

Studies have shown that cow’s milk and other milk-based products (e.g., cheeses) are potential routes of DON contamination [54,55]. Indirect sources, such as animal milk and various water reservoirs, were found to be intermittently contaminated, and human milk was routinely contaminated [50,55,56,57,58,59].

Winkler et al. (2015) developed a multi-toxin method for investigating the carryover of ZEA, DON, and its various metabolites from animal feed in cow’s milk [55]. They found that DON and its commonly considered nontoxic metabolite form, de-epoxy-DON (DOM-1), were present in a dose-dependent manner but at low doses (i.e., the highest levels were 0.250 µg/L and 0.56 µg/L, respectively). These results suggest that when DON is increased in animal feed, it can also be increased in milk but in its less toxic form (i.e., DOM-1). Other researchers have found similar results, and co-contaminant mycotoxins, such as ZEA and aflatoxin can also enter milk, albeit at low doses [50]. Aflatoxin M1 was the mycotoxin most frequently found in milk products, and it is also harmful to humans.

DON has also been found to persist in human milk and infant cereals. A study conducted in Turkey on the DON content in the breast milk of 90 new mothers found that DON was present in 100% of the samples, with a median occurrence of 3.9 µg/L (range 0.4–14.99 µg/L) [56]. Thirty-six percent of the breast milk samples contained values over the PMTDI recommendations [56]. An infant weighing 5 kg (i.e., 11 pounds) could easily consume more than two times the PMTDI (i.e., 2.4 µg/kg/bw/day) by consuming 800 mL of breast milk contaminated with DON at the highest levels of 14.99 µg/L. The exposure risk to infants was dependent on the dietary intake of each mother, and the authors emphasized that DON in breast milk could threaten infant health.

A US study showed that infant cereals were also contaminated, with 84% of samples displaying levels of 10–224 µg/kg (n = 52, 11 barley, 23 mixed grains, and 18 oats) [31,60]. However, while only 1.9% of samples exceeded European limits and none exceeded US limits, the assumption that the current limits protect children from the adverse effects of DON could be flawed [38]. Similarly, 66% of infant cereal samples in India were DON-positive, with levels ranging from 5 to 228 µg/kg (n = 29) [31].

To quantify infant risk from infant cereals, a 6-month-old weighing 7.9 kg fed a 30 g serving (approx. 1 ounce) at the European limit of 200 µg/kg would take in 0.8 µg/kg/bw. If fed only two servings daily, the child would quickly exceed the maximum PMTDI exposure levels (e.g., 1.6 µg/kg/bw/day). Thus, even low-level exposures can add up in small children. When combined with contaminated human milk, DON intake could quickly surpass the existing PMTDI guidelines.

Infant formula has also been recently studied for mycotoxin contamination. Forty-one baby formula samples were tested via ELISA in Chile, and 34% were contaminated with DON in a range of 17.07 µg/kg to 58.54 µg/kg [1]. If a 7-month-old infant weighing 8.3 kg consumed only 400 mL of formula per day at the highest level detected (i.e., 58.54), they would consume more than two times the PMTDI limit for DON (i.e., 2.8 µg/kg/bw/day). The same study also sampled infant cereals (n = 30) and found that 41% were contaminated with DON. Cereal contamination rates ranged from 30 µg/kg to 220 µg/kg DON at the upper limit [1]. Only one 30 g serving of cereal for the same 8.3 kg infant at 220 µg/kg brings them close to their PMTDI limit for the day at 0.8 µg/kg/bw/day. These levels are below the FDA regulation limit, demonstrating the importance of lowering the allowable levels in foods to protect infants.

DON has also been found in various water reservoirs in the US and internationally. In a study of 32 streams and 3 water treatment plants in Indiana, Iowa, and New York, DON was the most prevalent mycotoxin detected [57]. Of 116 samples taken, 77% of stream water samples were contaminated, presumably from crop and animal manure runoff, and 100% of human treatment plant wastewater effluent was contaminated [57]. During snowmelt in Iowa agricultural areas, the maximum levels were as high as 1.66 µg/L. Similar results were found in Switzerland in two earlier studies; however, DON levels were detected in smaller amounts, with the highest recorded level being 0.038 µg/L [58,61]. In addition, in a study of natural contaminants in the Douro River Estuary in Portugal, DON was again the most prevalent mycotoxin found in the water, with concentrations up to 0.374 µg/L. This is thought to result from runoff from crops, livestock effluent, and human wastewater from a local sewage treatment plant. This estuary supplied approximately 50% of the drinking water for the local urban population [59]. While these levels would not cause a child to surpass the PMTDI limits, they represent additive contributions to a child’s total toxin load.

Medicinal herbs and spices have also been found to be contaminated with mycotoxins and represent another additive increase in toxin exposure for infants and children when used to treat illness or spice foods [62]. Researchers in Korea found that multiple mycotoxins commonly contaminate these commodities. Samples from various countries were reviewed, including some from North America. Aflatoxins, ochratoxin A, fumonisins, ZEA, and DON were the most frequently detected mycotoxins in dried herbal medicines and spices. DON was detected in sage, chamomile, valerian root, senna, dried artichoke, dandelion, rhubarb, boldus, ginkgo, frangula, lemon verbena, olive leaves, red tea, white tea, spearmint, and star anise. The maximum DON levels varied by herb, ranging from 60 µg/kg to 321.2 µg/kg [62]. The authors attributed mycotoxin contamination to climate change, poor storage, insect damage, and infections at harvest.

Several other food commodities were found to be intermittently contaminated with DON. In a recent study conducted in China, which produces as much as 30% of the global rice supply, DON was present in 30.9% of the samples (n = 236) [63]. However, after processing by removing the husk and bran to produce white rice, the DON content was significantly lowered (e.g., the highest contamination levels were reduced from 2789 ± 301 µg/kg to 446 ± 64.8 µg/kg) [63]. However, even the lowered contamination levels exceeded the safe limit for infants and children. Rice cereal is a typical first baby food for US infants. For an 8-month-old infant weighing 8.6 kg, only one 30 g portion of rice cereal contaminated with 446 µg/kg DON will cause that infant to exceed the safe limits by taking in 1.6 µg/kg/bw/day.

Similarly, in a study conducted in Germany, it was shown that cocoa could become contaminated with DON. However, it was quickly mitigated by removing and processing the outer shell [43]. In the US, *Fusarium* species infections have also been found to cause potato rot, with levels in rotted tissue as high as 11,720 µg/L [64]. However, infections did not appear to penetrate the entire potato and could be mitigated by removing the rotten tissue within a 3 cm margin [64]. Whole nuts and seeds were also intermittently contaminated with DON, which depended more on storage conditions than crop infection [49]. The levels were also significantly lower than those found in cereal grains.

Many foods showed a low or limited risk of contamination with DON. Fresh fruits and vegetables, fresh herbs, honey, maple syrup, table sugar, and salt were not sources of DON. Likewise, dried fruits, fruit juices, and apple-based juices were not found to be sources of DON. However, they were often contaminated with other mycotoxins, such as patulin—another gastric irritant [65]. Tapioca, arrowroot, and coconut flours were not found to be regularly contaminated with DON. Aged cheeses and butter also did not appear to be significant sources of DON but could be sources of DOM-1 and other mycotoxins (e.g., aflatoxin M1 and ZEA) [50,55]. Fresh, unprocessed meats and eggs were also not found to be at risk of significant contamination with DON.

**Figure 2:**
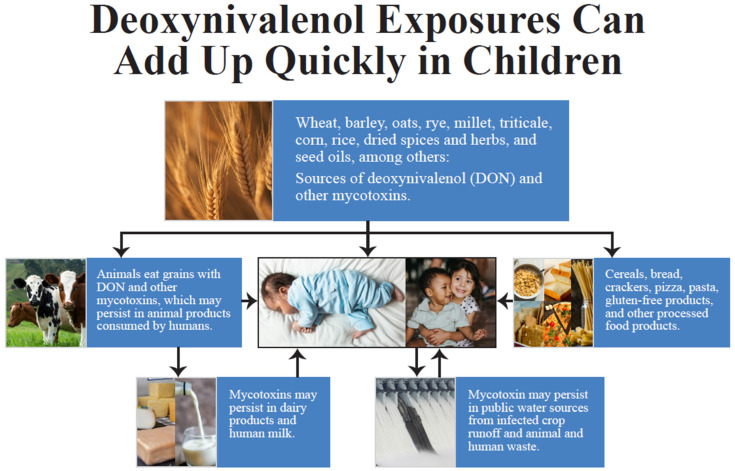
Sources of pediatric deoxynivalenol (DON) exposure.

Overall, DON is so prevalent that it appears ubiquitous in the food supply. Consequently, total avoidance might be unrealistic. Beyond grain-based products, DON has found its way into multiple commodities, including gluten-free foods, seed oils, nuts, tea, coffee, water reservoirs, and milk products, including human milk. Nevertheless, based on these data, mean DON levels in grains, such as wheat (878 ± 679 µg/kg), corn (1041 ± 1525 µg/kg), barley (2629 ± 1684 µg/kg), and oats (1359 ± 1882 µg/kg), still place children at the most significant risk of exceeding the safe limits of intake. All of these cereal grains exceeded the European safe limits, and all but wheat exceeded the US safe limits. Figure 2 provides a visual summary of the foods most commonly contaminated with DON and shows how repeated DON exposure from multiple commodities could add up quickly in children.

### 3.2. Assessing Pediatric Risk

The chronic ingestion of DON from multiple food commodities could adversely affect many body systems, including the intestinal microbiome [21]. Using the Brera et al. (2023) algorithm with the data collected here, a 26 kg child who eats 28 g (i.e., 1 ounce) of oats for breakfast contaminated at a mean level of 1359 µg/kg will consume 1464 ng/kg/bw/day (1.5 µg/kg/bw/day), exceeding the 1 µg/kg/bw/day PMTDI by breakfast [38]. If the same 26 kg child were to consume 56 g (i.e., 2 ounces) of noodles with 275 µg/kg DON at lunch, which is within the US guidelines, they would consume an additional 592 ng/kg/bw/day (i.e., 0.59 µg/kg/bw/day), and the child’s total DON intake would significantly exceed the PMTDI limit by lunch (i.e., 2.09 µg/kg/bw/day), placing that child at risk of adverse effects.

Because children eat significant quantities of cereal grains, their toxin load can increase quickly throughout the day. With added exposure from other commodities (e.g., moldy seed oils, tea, spices, nuts, and water), children are at increased risk of consuming exceedingly high amounts of DON. A child’s immature metabolic, immune, nervous, and gastrointestinal systems might not be equipped to handle such high toxic levels of exposure [16,17,66,67]. Furthermore, DON is typically accompanied by other mycotoxins (e.g., aflatoxin B1, ZEA, OTA, and fumonisins) that could further amplify its human health risks [10].

Based on the foodborne levels of DON identified here, chronic ingestion of DON from multiple food commodities might be common. With such potentially high pediatric exposure rates, DON’s drug-like effect on the gut microbiota, gastrointestinal tract, and immune and nervous systems should not be ignored. The following summarizes the potential impact of chronic DON exposure on various body systems at human exposure levels similar to those in this survey.

#### 3.2.1. Effects of DON on Intestinal Microbiota and Goblet Cell Integrity

The intestinal microbiota and mucus layer serve as a protective barrier and act as a first line of defense against numerous harmful microbes and xenobiotics, such as mycotoxins. DON has been found to interact with and alter intestinal microbial populations, dramatically affecting resident microbes and adversely affecting the integrity of the mucus barrier in animals and humans [21,22,23,24]. 

In a study on the effects of sub-chronic, low-level exposure to DON (e.g., 100 µg/kg) in human microbiota-transplanted rats, Saint-Cyr et al. (2013) found that exposure to DON changed the gut microbiota and increased the prevalence of Bacteroides species (*p* < 0.01) [23]. In a similar mouse model, DON was found to alter the intestinal microbiota, increasing the representation of Bacteroides over Firmicutes with high-dose exposure and significantly changing the microbiota at doses similar to those identified in the human food supply (e.g., 1000 µg/kg) [22]. For example, 79% of corn samples were contaminated at mean levels of 1041 ± 1525. Moreover, exposure to DON in mice caused the “perturbation of metabolic pathways, including phospholipid biosynthesis, sugar and poly-saccharide degradation, benzoate degradation, and ethylene biosynthesis”, among other processes (*p* < 0.05) [22]. 

Preliminary evidence shows that mucosal exposure to DON decreases the number of intestinal goblet cells available for mucus production in the human muco-secreting cell line HT29-16 at sub-toxic doses (e.g., 296 µg/L) (*p* < 0.01), decreasing the ability of goblet cells to produce mucins and formation of the protective mucus barrier [66,68]. Very low doses of DON (e.g., 296 µg/L) were also found to suppress trefoil messenger ribonucleic acid (mRNA) expression, inhibit trefoil secretion, and prevent wound healing in human and porcine cells (*p* < 0.01) [66]. Children are frequently exposed to levels above 296 µg/L, particularly from cereal grains, bakery products (e.g., 80% contaminated at 375 ± 306 µg/kg), and noodles (e.g., 89% contaminated at 275 ± 159 µg/kg). This level of exposure is also below the US regulatory guidance of 500 µg/kg.

The effects of DON’s acetylated derivatives on intestinal function are not considered in regulatory guidance. Pinton et al. (2012) demonstrated that exposing the intestinal cell line IPEC-1 with DON and feeding piglets chow contaminated with DON and two acetylated derivatives, 3-ADON and 15-ADON, caused intestinal histological changes, such as flattened and coalesced villi, disrupted tight junction and barrier functions, and interstitial edema (*p* < 0.05) [34]. The incidence and prevalence of pediatric celiac disease have been increasing across Europe with variations by region, which is hypothesized to have environmental underpinnings possibly related to high exposure to xenobiotics, such as DON [10,69]. To fully protect children from DON’s toxic effects, acetylated derivatives should be considered in regulatory guidelines.

#### 3.2.2. DON Exposure Increases Intestinal Permeability and the Translocation of Microbes

After DON penetrates the mucous barrier and microvilli, it can quickly diffuse through the intestinal mucosa and become bloodborne [15,34,70,71]. DON appears to affect TJs by binding to ribosomes and inhibiting the protein synthesis of claudin-4 proteins and possibly other claudin and TJ proteins at doses as low as 412 µg/L in human cell lines (*p* < 0.001), thereby decreasing adherens junction proteins and increasing intestinal permeability [72].

Increased intestinal permeability can then cause the increased uptake of DON and higher translocation rates of other luminal inhabitants and antigens, such as commensal and pathogenic bacteria (e.g., Mycobacterium and Yersinia), viruses, fungi, yeast, food antigens (e.g., gluten), and toxic chemicals [37,72]. This can then increase infection risk in humans and animals.

Vandenbroucke et al. (2009) demonstrated that DON exposure at doses as low as 250 µg/L increases the uptake of pathogenic Salmonella Typhimurium in porcine macrophages through the intestinal epithelium (*p* < 0.05) [73]. Vandenbroucke et al. (2011) subsequently demonstrated that with low-dose exposure to DON (e.g., 500–1000 µg/L), Salmonella Typhimurium was not only able to translocate across the intestinal epithelium in piglet jejunal cells more readily but also increased the inflammatory response to the infection (*p* < 0.05) [74]. Livestock are typically less sensitive to the effects of DON than humans. Therefore, the current contamination rates and levels should raise serious concerns about the safety of children in terms of increased infection risks with repeated exposure to DON.

#### 3.2.3. DON Triggers Immune Dysregulation and Increases Inflammation

The increased uptake of bacteria and other foreign antigens triggered by DON exposure can lead to a cascade-like inflammatory response that activates antigen-presenting cells and T-lymphocytes, activating the adaptive immune system [18,19,75,76,77]. Chung et al. (2003) found that murine macrophages subjected to doses as low as 100 µg/L induced tumor necrosis factor-alpha (TNF-α) and other cytokines through effects on mRNA-triggered p38 and extracellular signal-regulated kinase (ERK) mitogen-activated protein kinase (MAPK) pathways that regulate immune system cellular survival, differentiation, and growth (*p* < 0.05) [75]. Peska et al. (2005) also found that human Jurkat T cells subjected to low-level DON exposure (e.g., 250–1000 µg/L) exhibit inhibited protein synthesis and altered mRNA expression, with the induction of MAPK p38 pathways, which results in cellular apoptosis and adversely affects immune functions (*p* < 0.05) [19]. Thus, immune dysregulation can occur at typical dosages to which many children are exposed daily. These exposure levels are within the FDA guidelines for DON.

#### 3.2.4. DON Penetrates the BBB and Causes Neuronal Inflammation

DON intake can adversely affect the peripheral enteric nervous system by damaging and decreasing myenteric neurons and gliocytes [27]. Rissato et al. (2020) found that at typical intake levels considered safe for the public (e.g., 296 µg/L), the gliocyte cell area and neuronal cell body area of the myenteric plexus of the jejunum were reduced in Wistar rats (*p* < 0.05) [27]. Similarly, Razafimanjato et al. (2011) found that once DON becomes bloodborne, it can increase BBB permeability, affecting the central nervous system by decreasing the viability of glial cells, beginning at 1 µM (296 µg/L) (*p* < 0.05) [17].

Because of these detrimental effects, Razafimanjato et al. (2011) hypothesized that DON exposure might be an environmental variable influencing the pathogenesis of neurological diseases with glial involvement, such as ASD and Alzheimer’s disease [17]. Indeed, many children with ASD also suffer regularly from gastrointestinal distress that could be associated with the ingestion of toxicants such as DON [78]. 

#### 3.2.5. Nutritional Status and Growth

DON may affect a child’s health and nutritional status through multiple mechanisms. DON appears to adversely influence nutrient absorption through active uptake via sodium glucose-dependent transport 1 (SGLT1). By affecting SGLT1, DON can impair glucose uptake, leading to an osmotic gradient, diarrhea, and the malabsorption of water and other nutrients [5,79]. Facilitated glucose transport is another transcellular pathway that could be altered by DON uptake via glucose transporter 2 (GLUT2) [5,79]. At DON levels as low as 296 µg/L, amino acid and sugar uptake in the human intestinal cell line HT-29-D4 was adversely affected (*p* < 0.01) [5]. Conversely, saturated lipid uptake (e.g., palmitate) appeared to markedly increase at a realistic, somewhat higher human exposure dose of 10 µM (2963 µg/L) [5]. In this survey, 48% of the barley samples were contaminated at these levels (e.g., 2629 ± 1684 µg/kg).

Overall, the global DON exposure rates identified here are similar to the rates found to cause harm in animals and humans. Because of the potential for DON to cause harm, Brera et al. (2023) recently suggested that the maximum levels in foods intended for infants and children should be set below 200 µg/kg of DON, as initially suggested by Pieters et al. (2002) [30,38]. Unfortunately, based on global trends toward global warming and the increased prevalence of this toxicant within the food chain, DON contamination rates below 200 µg/kg will be difficult to achieve [13].

### 3.3. Global Spread of Fusarium Infections

The *Fusarium* fungi that produce trichothecene mycotoxins, such as DON, were initially found to thrive in the cool, wet climates of Northern Europe, North America, Canada, Russia, and China, among other temperate climates, and grow best between 0 °C (32 °F) and 25 °C (77 °F). *Fusarium* species crop infections have since spread to newly developing countries and have become a global problem for various agricultural reasons. For example, countries such as those in Africa now regularly grow wheat, corn, and other grains that potentially harbor *Fusarium* infections [31].

The Food and Agriculture Organization of the United Nations and the WHO have reviewed global agricultural and food processing practices related to mycotoxin contamination and mycotoxins such as DON in food commodities as potential public health hazards. Reasons for increased fungal infections in crops that lead to mycotoxin contamination include climate change, inappropriate crop rotation between infected plant species (e.g., wheat and corn) instead of rotating crops with infection-resistant species, reduced tilling practices, and insect damage, among other variables [12]. Good farming practices are essential to preventing mycotoxin contamination but might not completely control the problem owing to climate change. In a 15-year study in Croatia and Serbia, climate shifts, including temperature, precipitation, and humidity, increased the DON occurrence in various commodities, particularly cereal grains [80]. In addition, pesticides and fungicides have done little to control their occurrence and can have toxic effects on humans [12]. Furthermore, some fungicides might encourage *Fusarium* growth by stimulating mycotoxin production through chemical–pathogen interactions that are not well understood [81,82]. For example, strobilurin fungicides have been found to increase DON levels by 6–18% in harvested grain after treatment [82]. Mycotoxin-degrading bacteria could be a promising alternative to toxic chemicals for fungal infection management. Many microbes have also been found to detoxify trichothecene mycotoxins, including DON, such as *Lactobacillus rhamnosus*; specific *Bacillus* (*B.*) species, such as *B. subtilis*, *B. coagulans*, and *B. velezensis*; and *Clostridium* species WJ06, to varying degrees, and most have been proven to be safe for human consumption [83,84,85,86,87,88].

After harvesting, food processing practices can also influence fungal growth. Fungal spores that cause food contamination are found not only in soil and residues from infected crops but also in farming and food processing equipment and within storage structures, despite diligent cleaning [12]. Factors that affect spore germination involve environmental and storage issues related to humidity and temperature [12,36]. Humidity and temperature variables that promote mold growth in food can vary from region to region, year to year, season to season, and storage facility to storage facility. In large storage facilities with limited climate control technology, precise temperature and moisture monitoring of stored grains and other commodities might not be possible. Moreover, some *Fusarium* species are remarkably tenacious and require 20% or lower humidity to control their growth, which is unlikely to occur in most storage facilities [89]. Once present in foods, DON is stable at high temperatures and under acidic conditions, making it nearly impossible to eliminate through processing [31,90,91]. This problem could be even further intensified when grains or other food commodities awaiting production are stored for extended periods due to the food and food processing needs in various regions, allowing for a wide variety of contaminated foods to enter the global marketplace unchecked [12].

Thus, farming, food processing practices, and climate-related variables can enhance growth environments for toxigenic mold species, increasing mycotoxin contamination in foods. Because of all these variables, some believe that there might be no way of preventing human mycotoxin exposure from processed foods, making them among the biggest threats to the human food supply, especially to vulnerable children [12,92]. Despite these assertions, solutions are urgently needed to protect infants and children.

### 3.4. Mycotoxin Monitoring to Protect Children

Humans cannot see, taste, or smell mycotoxins in foods to avoid them. Consequently, tracking mycotoxins, such as DON, to ensure food safety and protect vulnerable youth requires specialized equipment and can be expensive. DON and other mycotoxins can be tracked through direct measurements in foods and beverages or through indirect urinary analysis methods to verify their presence [9,35,93,94]. Direct methods (e.g., lateral flow technology, LC-MS/MS) require potentially contaminated food ingredients to be tested and contaminated ones to be discarded before selling them to the public. Indirect methods (e.g., urinalysis) can detect DON consumption in individuals. However, they can only identify the specific food source if measurements are taken before and after eliminating a suspected food item.

Using indirect methods, one study in the United Kingdom (UK) demonstrated that by eliminating wheat intake, urinary measures of DON in humans decreased from 7.2 ng DON/mg creatinine (95% confidence interval (CI) 4.5–10.5 ng/mg) to 0.6 ng DON/mg creatinine (*p* < 0.001; 95% CI 0.4–0.9 ng/mg) over 48 h [35]. However, eliminating only wheat might not be an effective mitigation strategy in regions where corn, barley, rice, gluten-free bakery products, tea, and water, among other commodities, harbor mycotoxin-producing fungi.

Other studies using urinary analysis coupled with food frequency analysis have helped to verify that young children tend to consume higher levels of DON per kilogram of body weight than adults owing to the types of foods frequently eaten relative to their body size (e.g., wheat- and corn-based cereals, pasta, and bread) [31]. Furthermore, some studies involving children have verified intake levels greater than two times the PMTDI limit [9,31].

Genetic differences among children can also influence their urine DON levels, as was seen in a study comparing children with the neurological disability ASD to their non-ASD siblings and controls [25]. De Santis et al. (2017) found significant differences in urinary DON and DOM1 levels between ASD and non-ASD siblings (*p* = 0.0141 and *p* = 0.0259, respectively) [25]. They also found differences in aflatoxin M1, ochratoxin A, and fumonisin B1. Interestingly, DON, ochratoxin A, and fumonisins are frequently found in grains, and aflatoxin M1 and DOM1 are found in milk products. Many parents have subjectively found that their children with ASD perform better on grain-free, dairy-free diets, which suggests a need for a deeper examination of the role of foodborne mycotoxins in the etiology of this disorder.

Serum levels of DON are not typically tracked because it is rapidly cleared from the bloodstream. However, tracking it might be warranted to help identify metabolic and genetic differences in children that could contribute to the development of diseases such as ASD [4,26]. Additionally, a child’s ability to detoxify and eliminate harmful mycotoxins such as DON could also be influenced by their gut microbiota, which has recently been a topic of great interest [21,22,23,95].

### 3.5. Gut Microbiota, Mycotoxins, and Detoxification

The gut microbiota is believed to act as an extra-organ system that plays an essential role in the maturation of the intestinal mucosa and immune system [67]. Gut-dwelling organisms in humans appear to be involved in many physiological functions that help detoxify chemicals, metabolize nutrients, produce short-chain fatty acids that protect the gut, and influence the immune system [67,95].

Infants are born with extraordinarily little microbiome diversity, although Candida fungal species appear to colonize infants from the mother’s microbiota during vaginal birth [96]. Bacteria, fungi, and other inhabitants are steadily increased in the human gut as children age due to various environmental exposures. The human mouth provides the most significant amount of exposure throughout life because it allows for the direct entry of microorganisms into the gastrointestinal tract [96]. An estimated 100 trillion microbes are believed to reside in the human gut by adulthood; these tiny organisms can outnumber human cells by more than 10 to 1 [96].

The human diet might have once enhanced the landscape of the human microbiome. For millennia, humans ate plants (e.g., raw vegetables, grasses, tubers, and fruits), which can be rich sources of live dietary microbes that help build and maintain a healthy microbiome. Westernized diets containing added sugars, fat, salt, bacteria-killing preservatives, and additives—and also rich in fungal contaminants such as DON—could have begun to diminish and change the human microbiome, potentially leaving humans less capable of detoxifying dietary contaminants [97]. Indeed, animals with a high gut bacterial content (e.g., ruminants and poultry) are better protected from pathogenic microorganisms and hazardous xenobiotics such as DON and its derivatives [67].

The bacterial detoxification of mycotoxins appears to be an essential function of some bacterial species that, like fungi, produce secondary metabolites that inactivate and, at times, completely degrade mycotoxins such as DON [83,84,85,86,87,88]. However, young children with immature microbiomes might not have sufficient microbial defenses to detoxify DON—certainly not at the exceedingly high doses prevalent in today’s food supply.

Agricultural communities have long been aware that mycotoxins such as DON negatively affect livestock growth and development, cause immune dysregulation and intestinal inflammation, and can lead to increased enteric infection rates [18,72,73,74,77,79,98,99]. Farming communities have learned to mitigate the adverse effects of biohazards such as DON by adding targeted mycotoxin binders and probiotics to contaminated feed. At present, there is no such mitigation strategy for the human food supply other than to limit exposure, which, thus far, appears to be ineffective in protecting children.

A better understanding of the interplay between microbial species in the micro- and mycobiomes of humans is needed to develop an effective mitigation plan to protect vulnerable youth. A wealth of chemical information could be gleaned for large-scale mycotoxin detoxification efforts by studying the microbial interplay between species and locating human-friendly species to be used in the microbial degradation of mycotoxins [100].

### 3.6. Implications for Healthcare Practice and Policy

Numerous nutritional strategies have been developed by healthcare professionals aimed at improving child health and decreasing foodborne illness. However, mycotoxins rarely find their way into healthcare discussions unless a large outbreak of mycotoxicosis is discovered only after other pathogens are ruled out. Instead, mycotoxins have been studied for pharmaceutical purposes. Indeed, many mold-borne secondary metabolites have been used to create medications, including penicillin, cyclosporin, and statins, among many other life-saving medicines [92]. Regardless, mycotoxins such as DON have no medicinal purpose and can cause considerable illness after repeated exposure to contaminated food [31,92].

Based on the data reviewed in this study, it is not a matter of if young patients will be exposed to harmful toxins such as DON but rather to how much they will be exposed. It is unclear whether the common occurrence of mycotoxin contamination in food and drinks plays a major or minor role in the microbial perturbations, growth stunting, intestinal distress, and neurological impairments experienced by some children [10,12,26,101]. However, it is clear that limiting mycotoxin exposure by avoiding contaminated grains and other commodities could help limit pediatric exposure to this toxicant. A seemingly simple answer to this problem would be to increase children’s intake of unprocessed whole foods (e.g., fresh fruits, vegetables, and herbs). This would help to decrease the total proportion of contaminated foods eaten by children. Furthermore, eating mycotoxin-contaminated foods in a mixed meal with high-nutrient-density, non-contaminated foods could reduce their absorption, resulting in less toxicity [102]. Unfortunately, such recommendations often go unheeded by the public. Fresh, whole fruits and vegetables, among other healthful choices, appear to be no match for the allure of well-advertised ultra-processed foods that promise great taste with limited nutritional value [103,104].

To further exacerbate the difficulty children and families might have with dietary changes, children who are required to follow interventional diets (e.g., those with celiac disease) have demonstrated that having to eat differently than their peers can lead to feelings of isolation to which many might not be amenable [105,106]. Historically, pediatric populations with dietary constraints, such as those with celiac disease, have demonstrated that very restrictive diets that do not consider food preferences are not only difficult to follow but also can negatively affect social and emotional well-being and might undermine children’s quality of life [105,106].

Thus, having children eat differently from their peers might not be an effective mitigation plan. Considering that children today eat many wheat-based products (e.g., bread, pasta, crackers, biscuits, and cakes), Brera et al. (2023) attempted to address this problem by developing a “low DON” wheat flour [38]. Their methods involved carefully selecting wheat suppliers, maintaining correct transport, implementing proper storage conditions of raw materials and final products, preventing production process alterations, and predetermining safe contamination levels in final products [38]. Despite these measures, it was not possible to eliminate DON from flour. DON levels in the lowest-level cooking flours were reduced from mean levels of 358.9 ± 4.1 µg/kg to 172.3 ± 4.4 µg/kg [38]. Brera et al. (2023) also noted that at levels higher than this, repeated exposure in children resulted in intake levels above the PMTDI [38]. Thus, the current legal limits for DON in foods did not guarantee child safety, suggesting that tighter regulations are needed to protect vulnerable youth. The US FDA permissible levels are more than double those set in Europe, potentially placing American children at an even greater risk [33].

Brera et al. (2023) also emphasized the importance of clearly communicating to consumers, transparently and truthfully, the levels of these contaminants in foods out of respect for their right to know and right to choose in the marketplace [38]. Indeed, increasing public awareness regarding the presence of DON in foods frequently eaten by children and their effects on the human body might prompt caregivers to make healthier choices for youth (e.g., fresh fruits and vegetables).

A feasible mitigation plan to protect vulnerable children could involve stricter government guidelines for mold toxin levels in foods, including more stringent oversight of food processors to safeguard all children. Developing a “low mycotoxin diet” that provides acceptable alternatives to existing grain-based products (e.g., bread, pasta, and baked goods) using non-grain-based flours that are less likely to be contaminated with DON (e.g., tapioca flour and coconut flour) could also provide a temporary solution, but such alternatives would still require close monitoring for contaminants that place children at risk. Using microbes that are safe for human consumption to detoxify mycotoxins in the field and during food processing might also be an effective solution in the future. To mitigate this problem adequately, global efforts involving teams of healthcare providers, scientists, farmers, food processors, and policymakers, among other key stakeholders, are needed to develop effective strategies to protect youth from this harmful toxicant.

### 3.7. Limitations

This integrated review is limited by the heterogeneity of the methods used in various studies to detect mycotoxin exposure in food commodities. The studies sourced for this review used various testing methods, such as LC-MS/MS, high-performance liquid chromatography, ELISA testing, and Veratox Fast kits. While all the selected studies used validated methods for detection, some methods of detection are more sensitive than others. To add value to this body of knowledge and better quantify pediatric risk, researchers should attempt to quantify the presence of DON and other mycotoxins in food commodities using a more standardized and precise approach (e.g., LC-MS/MS) [107]. Another limitation was that adults exposed to high DON levels, such as vegan vegetarians and those living in farming areas, among other vulnerable groups, were not discussed. Lastly, the US is underrepresented in the data presented here, owing to a lack of available studies.

### 3.8. Implications for Future Research

More research is needed in the US to explore contamination rates in foods frequently eaten by children. Packaged cereals, pasta, pizza, dairy products, and even drinking water should be assessed to help gauge the magnitude of mycotoxin prevalence in these commodities and provide data to develop a mitigation plan to protect vulnerable youth.

Future research using validated urinary markers could also help to clarify the extent to which young patients are exposed to mycotoxins, exceed the PMTDI limit, and subsequently suffer adverse effects. Studies on the effect of DON on humans and its influence on the development of chronic diseases based on natural exposure levels will also be needed for policymakers to establish optimal regulatory guidance levels for children.

To better understand the relationship between exposure to DON and the human microbiome, well-controlled clinical trials focusing on lowering dietary mycotoxins with a simultaneous analysis of their effects on the microbiome should be designed. Identifying the role of fungal secondary metabolites such as DON and other chemical mediators involved in inter- and intra-microbial communication systems in humans could lead to new treatment options to control the effects of their regular presence in foods.

## 4. Conclusions

On a global scale, the routine contamination of cereal grains, bakery products, pasta, and human milk with DON could lead to intake levels above PMTDI limits. Other DON-contaminated food commodities, such as soy, coffee, tea, dried spices, nuts, certain seed oils, animal milk, and various water reservoirs, can also incrementally increase a child’s toxin load. Such cumulative exposure to DON from such widespread contamination can easily result in DON intake exceeding the PMTDI limits, leading to harmful effects on infants’ and children’s gastrointestinal tracts, immune and nervous systems, and microbiomes. Better mitigation strategies and global measures to improve farming and food processing practices are needed to safeguard vulnerable youth from this harmful toxicant. Global efforts that include tighter regulations and teams of healthcare providers, scientists, public health experts, farmers, food processors, and policymakers, among other key stakeholders, are needed to develop effective strategies that better protect infants and children.

## Figures and Tables

**Table 1:** Exposure limits set in Europe and by the US FDA for deoxynivalenol.

DON’s Effects on Humans	Fungal Species	Food Commodity	US FDA (µg/kg)	Europe (µg/kg)
Targets actively dividing cells, such as those lining the GI tract and skin, lymphoid, and erythroid cells. Esophageal cancer. Categorized as a “ribotoxin” and protein synthesis inhibitor.	*Fusarium graminearum*; *Fusarium culmorum*	Cereals, cereal products, maize, wheat, barley, rye, buckwheat, oats, millet, triticale, rice, sorghum, alcoholic beverages made from cereal grains	1000 in finished wheat products, such as flour, bran, and germ; 500 in cereal-based foods for infants and children	200–500 in processed grains; 200 in products for infants

Sources for the table: [31,33,35,36,37].

**Table 2:** Deoxynivalenol (DON) contamination in the global food supply.

Crop/Food Tested and Reference(s)	Countries Represented	Total Number of Studies (N)	Total Sample Size (n)	Percentage Positive Samples (%)	Mean and Standard Deviation in µg/kg or µg/L (Liquids)
Wheat [31,41]	Argentina, Brazil, Canada, China, India, Iran, Israel, Italy, Netherlands, Serbia, Romania, Uruguay, Japan, Sweden, Slovakia, Finland, Hungary, Switzerland, Poland, Nigeria, Spain	33	4520	89%	878 ± 679 µg/kg
Maize (corn) [31,41]	Hungary, Portugal, Serbia, Japan, South Africa	7	615	79%	1041 ± 1525 µg/kg
Barley [31,41]	Argentina, Brazil, Romania, Japan, Tunisia	6	386	48%	2629 ± 1684 µg/kg
Bakery products [31]	Brazil, Hungary, Serbia,	3	398	80%	375 ± 306 µg/kg
Noodles and pasta [31]	Italy, Germany	2	67	89%	275 ± 159 µg/kg
Oats [31]	Finland, Sweden, United Kingdom	3	427	50%	1359 ± 1882 µg/kg
Rice [41]	Japan	1	60	40%	18 ± NA µg/kg
Soy [48]	Germany	1	45	13%	67.2 ± 96.2 µg/kg
Azuki beans [41]	Japan	1	40	38%	12 ± NA µg/kg
Nuts [49]	Portugal	1	14 (Almonds) 3 (Cashews) 7 (Hazelnuts)	36% 100% 86%	2.85 ± NA µg/kg 135.8 ± NA µg/kg 336.5 ± NA µg/kg
Cow’s milk [50]	Argentina	1	704	40%	0.388 ± NA µg/L
Vegetarian milks [51]	Spain	1	8 Oat milk 8 Soy milk 5 Rice milk 1 Nut milk	38% 0% 0% 0%	22.7 ± 3.99 µg/L
Coffee [45]	Spain	1	169	43%	67 ± NA µg/kg
Tea [42,52,53]	China (Pu-Erh tea) Latvia (Herbal tea) Lebanon (Kaak tea)	1 1 1	70 60 20	90% 45% 50%	1952.2 ± 646 µg/kg 1198 ± NA µg/L 70 + NA g/kg
Seed oils (rapeseed and linseed) [40]	Lithuania Rapeseed Linseed	1	62 32	75% 100%	12.46 ± 7.81 µg/kg 25.06 ± 12.13 µg/kg

Mean data from various studies were combined to calculate the average incidences of contamination in samples. Contamination rates were found to vary from month to month, product to product, and region to region, depending on the weather conditions, storage, and food processing circumstances, among other factors. NA= No available standard deviation data.

## Data Availability

All data retrieved for this study are included in table or narrative format within the body of the paper.

## References

[1] Foerster C., Monsalve L., Rios-Gajardo G. (2024). Infant Exposure to Ochratoxin A, Zearalenone, and Deoxynivalenol from the Consumption of Milk Formula and Baby Cereal in Chile. Food Res. Int..

[2] Bennett J.W., Klich M. (2003). Mycotoxins. Clin. Microbiol. Rev..

[3] Kankkunen P., Rintahaka J., Aalto A., Leino M., Majuri M., Alenius H., Wolff H., Matikainen S. (2009). Trichothecene Mycotoxins Activate Inflammatory Response in Human Macrophages. J. Immunol..

[4] Notenboom S., Hoogenveen R.T., Zeilmaker M.J., Van den Brand A.D., Assuncao R., Mengelers M.J.B. (2023). Development of a Generic PBK Model for Human Biomonitoring with an Application to Deoxynivalenol. Toxins.

[5] Maresca M., Mahfoud R., Garmy N., Fantini J. (2002). The Mycotoxin Deoxynivalenol Affects Nutrient Absorption in Human Intestinal Epithelial Cells. J. Nutr..

[6] Milicevic D., Nastasijevic I., Petrovic Z. (2016). Mycotoxin in the Food Supply Chain-Implications for Public Health Program. J. Environ. Sci. Health. C. Environ. Carcinog. Ecotoxicol. Rev..

[7] Altomare C., Logrieco A.F., Galio A. (2021). Mycotoxins and mycotoxigenic fungi: Risk Management. A challenge for future global food safety. Encyclopedia of Mycology.

[8] Zain M.Z. (2010). Impact of Mycotoxins on Humans and Animals. J. Saudi Chem. Soc..

[9] Papageorgiou M., Wells L., Williams C., White K., De Santis B., Liu Y., Debegnach F., Miano B., Moretti G., Greetham S. (2018). Assessment of Urinary Deoxynivalenol Biomarkers in UK Children and Adolescents. Toxins.

[10] Maresca M., Fantini J. (2010). Some Food-Associated Mycotoxins as Potential Risk Factors in Humans Predisposed to Chronic Intestinal Inflammatory Diseases. Toxicon.

[11] Pestka J.J. (2010). Deoxynivalenol: Mechanisms of Action, Human Exposure, and Toxicological Relevance. Arch. Toxicol..

[12] Food and Agricultural Organization of the United Nations, World Health Organization Code of Practice for the Prevention and Reduction of Mycotoxin Contamination in Cereals (CXC 51-2003). *Codex Alimentarius International Food Standards*. 2016, Rome, Italy, 1–16. https://www.fao.org/fao-who-codexalimentarius/sh-proxy/ru/?lnk=1&url=https%253A%252F%252Fworkspace.fao.org%252Fsites%252Fcodex%252FStandards%252FCXC%2B51-2003%252FCXC_051e.pdf.

[13] Li F., Duan X., Zhang L., Jiang D., Zhao X., Meng E., Yi R., Liu C., Li Y., Wang J. (2022). Mycotoxin Surveillance on Wheats in Shandong Province, China, Reveals Non-Negligible Probabilistic Health Risk of Chronic Gastrointestinal Diseases Posed by Deoxynivalenol. Environ. Sci. Pollut. Res. Int..

[14] Flannery B.M., Clark E.S., Pestka J.J. (2012). Anorexia Induction by the Trichothecene Deoxynivalenol (Vomitoxin) is Mediated by the Release of the Gut Satiety Hormone Peptide YY. Toxicol. Sci..

[15] Pestka J.J. (2008). Mechanisms of Deoxynivalenol-Induced Gene Expression and Apoptosis. Food Addit Contam. Part A Chem. Anal. Control. Expo. Risk Assess..

[16] Zhang J., You L., Wu W., Wang X., Chrienova Z., Nepovimova E., Wu Q., Kuca K. (2020). The Neurotoxicity of Trichothecenes T-2 Toxin and Deoxynivalenol (DON): Current Status and Future Perspectives. Food Chem. Toxicol..

[17] Razafimanjato H., Benzaria A., Taieb N., Guo X., Vidal N., Di Scala C., Varini K., Maresca M. (2011). The Ribotoxin Deoxynivalenol Affects the Viability and Functions of Glial Cells. Glia.

[18] Cano P.M., Seeboth J., Meurens F., Cognie J., Abrami R., Oswald I.P., Guzylack-Piriou L. (2013). Deoxynivalenol as a New Factor in the Persistence of Intestinal Inflammatory Diseases: An Emerging Hypothesis through Possible Modulation of Th17-Mediated Response. PLoS ONE.

[19] Pestka J.J., Uzarski R.L., Islam Z. (2005). Induction of Apoptosis and Cytokine Production in the Jurkat Human T Cells by Deoxynivalenol: Role of Mitogen-Activated Protein Kinases and Comparison to Other 8-Ketotrichothecenes. Toxicology.

[20] Yang H., Chung D.H., Kim Y.B., Choi Y.H., Moon Y. (2008). Ribotoxic Mycotoxin Deoxynivalenol Induces G2/M Cell Cycle Arrest Via p21Cip/WAF1 mRNA Stabilization in Human Epithelial Cells. Toxicology.

[21] Jin J., Zhang C., Ren X., Tai B., Xing F. (2023). Metagenome Analysis Identifies Microbial Shifts upon Deoxynivalenol Exposure and Post-Exposure Recovery in the Mouse Gut. Toxins.

[22] Wang J., Zhang R., Zhai Q., Liu J., Li N., Liu W., Li L., Shen W. (2019). Metagenomic Analysis of Gut Microbiota Alteration in a Mouse Model Exposed to Mycotoxin Deoxynivalenol. Toxicol. Appl. Pharmacol..

[23] Saint-Cyr M.J., Perrin-Guyomard A., Houee P., Rolland J.G., Laurentie M. (2013). Evaluation of an Oral Subchronic Exposure of Deoxynivalenol on the Composition of Human Gut Microbiota in a Model of Human Microbiota-Associated Rats. PLoS ONE.

[24] Bracarense A.P., Lucioli J., Grenier B., Drociunas Pacheco G., Moll W.D., Schatzmayr G., Oswald I.P. (2012). Chronic Ingestion of Deoxynivalenol and Fumonisin, Alone Or in Interaction, Induces Morphological and Immunological Changes in the Intestine of Piglets. Br. J. Nutr..

[25] De Santis B., Raggi M.E., Moretti G., Facchiano F., Mezzelani A., Villa L., Bonfanti A., Campioni A., Rossi S., Camposeo S. (2017). Study on the Association among Mycotoxins and Other Variables in Children with Autism. Toxins.

[26] De Santis B., Brera C., Mezzelani A., Soricelli S., Ciceri F., Moretti G., Debegnach F., Bonaglia M.C., Villa L., Molteni M. (2019). Role of Mycotoxins in the Pathobiology of Autism: A First Evidence. Nutr. Neurosci..

[27] Rissato D.F., de Santi Rampazzo A.P., Borges S.C., Sousa F.C., Busso C., Buttow N.C., Natali M.R.M. (2020). Chronic Ingestion of Deoxynivalenol-Contaminated Diet Dose-Dependently Decreases the Area of Myenteric Neurons and Gliocytes of Rats. Neurogastroenterol. Motil..

[28] Gromadzka K., Pankiewicz J., Beszterda M., Paczkowska M., Nowakowska B., Kocylowski R. (2021). The Presence of Mycotoxins in Human Amniotic Fluid. Toxins.

[29] Tan T., Chen T., Zhu W., Gong L., Yan Y., Li Q., Chen L., Li Y., Liu J., Li Y. (2023). Adverse Associations between Maternal Deoxynivalenol Exposure and Birth Outcomes: A Prospective Cohort Study in China. BMC Med..

[30] Pieters M.N., Freijer J., Baars B., Fiolet D.C.M., van Klaveren J., Slob W. (2002). Risk Assessment of Deoxynivalenol in Food: Concentration Limits, Exposure and Effects. Adv. Exp. Med. Biol..

[31] Mishra S., Srivastava S., Dewangan J., Divakar A., Kumar Rath S. (2019). Global Occurrence of Deoxynivalenol in Food Commodities and Exposure Risk Assessment in Humans in the Last Decade: A Survey. Crit. Rev. Food Sci. Nutr..

[32] Food and Agricultural Organization of the United Nations, World Health Organization Codex Alimentarius International Food Standards CXS 193-1995. 1995, Rome, Italy, 43–47. https://www.fao.org/fao-who-codexalimentarius/sh-proxy/es/?lnk=1&url=https%253A%252F%252Fworkspace.fao.org%252Fsites%252Fcodex%252FStandards%252FCXS%2B193-1995%252FCXS_193e.pdf.

[33] Food and Drug Administration Guidance for Industry and FDA: Advisory Levels for Deoxynivalenol (DON) in Finished Wheat Products for Human Consumption and Grains and Grain by-Products used for Animal Feed. 2010, Rockville, MD, *FDA-2013-S-0610*. https://www.fda.gov/regulatory-information/search-fda-guidance-documents/guidance-industry-and-fda-advisory-levels-deoxynivalenol-don-finished-wheat-products-human.

[34] Pinton P., Tsybulskyy D., Lucioli J., Laffitte J., Callu P., Lyazhri F., Grosjean F., Bracarense A.P., Kolf-Clauw M., Oswald I.P. (2012). Toxicity of Deoxynivalenol and its Acetylated Derivatives on the Intestine: Differential Effects on Morphology, Barrier Function, Tight Junction Proteins, and Mitogen-Activated Protein Kinases. Toxicol. Sci..

[35] Turner P.C., Burley V.J., Rothwell J.A., White K.L., Cade J.E., Wild C.P. (2008). Dietary Wheat Reduction Decreases the Level of Urinary Deoxynivalenol in UK Adults. J. Expo. Sci. Environ. Epidemiol..

[36] Alshannaq A., Yu J.H. (2017). Occurrence, Toxicity, and Analysis of Major Mycotoxins in Food. Int. J. Environ. Res. Public. Health..

[37] Park S.H., Kim D., Kim J., Moon Y. (2015). Effects of Mycotoxins on Mucosal Microbial Infection and Related Pathogenesis. Toxins.

[38] Brera C., De Santis C., Marzona S., Gregori E., Prisco S.S., Monti M., Chilosi G., Pantanali A. (2023). Exposure Assessment to Deoxynivalenol of Children Over 3 Years Deriving from the Consumption of Processed Wheat-Based Products Produced from a Dedicated Flour. Toxins.

[39] Centers for Disease Control and Prevention (2009). CDC Growth Charts: United States. https://www.cdc.gov/growthcharts/background.htm.

[40] Mankeviciene A., Suproniene S., Brazauskiene I., Gruzdeviene E. (2011). Natural Occurrence of *Fusarium* Mycotoxins in Oil Crop Seed. Plant Breed. Seed Sci..

[41] Yoshinari T., Takeuchi H., Aoyama K., Taniguchi M., Hashiguchi S., Kai S., Ogiso M., Sato T., Akiyama Y., Nakajima M. (2014). Occurrence of Four *Fusarium* Mycotoxins, Deoxynivalenol, Zearalenone, T-2 Toxin, and HT-2 Toxin, in Wheat, Barley, and Japanese Retail Food. J. Food Prot..

[42] Wu J., Yang G., Chen J., Li W., Li J., Fu C., Jiang G., Zhu W. (2014). Investigation for Pu-Erh Tea Contamination Caused by Mycotoxins in a Tea Market in Guangzhou. J. Basic Appl. Sci..

[43] Raters M., Matissek R. (2007). Sensitive Method for Determination of DON in Cocoa by Means of HPLC-Techniques. Mycotoxin Res..

[44] Lee T., Lee S.H., Lee S.H., Shin J.Y., Yun J.C., Lee Y.W., Ryu J.G. (2011). Occurrence of *Fusarium* Mycotoxins in Rice and its Milling by-Products in Korea. J. Food Prot..

[45] Garcia-Moraleja A., Font G., Manes J., Ferrer E. (2015). Analysis of Mycotoxins in Coffee and Risk Assessment in Spanish Adolescents and Adults. Food Chem. Toxicol..

[46] Schollenberger M., Muller H.M., Rufle M., Suchy S., Planck S., Drochner W. (2005). Survey of *Fusarium* Toxins in Foodstuffs of Plant Origin Marketed in Germany. Int. J. Food Microbiol..

[47] Yuan Q., Yang P., Wu A., Zuo D., He W., Guo M., Huang T., Li H., Liao Y. (2018). Variation in the Microbiome, Trichothecenes, and Aflatoxins in Stored Wheat Grains in Wuhan, China. Toxins.

[48] Schollenberger M., Muller H.M., Rufle M., Terry-Jara H., Suchy S., Plank S., Drochner W. (2007). Natural Occurrence of *Fusarium* Toxins in Soy Food Marketed in Germany. Int. J. Food Microbiol..

[49] Cunha S.C., Sa S.V.M., Fernandes J.O. (2018). Multiple Mycotoxin Analysis in Nut Products: Occurrence and Risk Characterization. Food Chem. Toxicol..

[50] Signorini M.L., Gaggiotti M., Molineri A., Chiericatti C.A., Zapata de Basilico M.L., Basilico J.C., Pisani M. (2012). Exposure Assessment of Mycotoxins in Cow’s Milk in Argentina. Food Chem. Toxicol..

[51] Hamed A.M., Arroyo-Manzanares N., Garcia-Campana A.M., Gamiz-Gracia L. (2017). Determination of *Fusarium* Toxins in Functional Vegetable Milks Applying Salting-Out-Assisted Liquid-Liquid Extraction Combined with Ultra-High-Performance Liquid Chromatography Tandem Mass Spectrometry. Food Addit Contam. Part A Chem. Anal. Control. Expo. Risk Assess..

[52] Reinholds I., Bogdanova E., Pugajeva I., Bartkevics V. (2019). Mycotoxins in Herbal Teas Marketed in Latvia and Dietary Exposure Assessment. Food Addit Contam. Part B Surveill..

[53] Soubra L., Sarkis D., Hilan C., Verger P. (2009). Occurrence of Total Aflatoxins, Ochratoxin A and Deoxynivalenol in Foodstuffs Available on the Lebanese Market and their Impact on Dietary Exposure of Children and Teenagers in Beirut. Food Addit Contam. Part A Chem. Anal. Control. Expo. Risk Assess..

[54] Swanson S.P., Dahlem A.M., Rood H.D., Cote L.M., Buck W.B., Yoshizawa T. (1986). Gas Chromatographic Analysis of Milk for Deoxynivalenol and its Metabolite DOM-1. J. Assoc. Off. Anal. Chem..

[55] Winkler J., Kersten S., Valenta H., Meyer U., Engelhardt U.H., Danicke S. (2015). Development of a Multi-Toxin Method for Investigating the Carryover of Zearalenone, Deoxynivalenol and their Metabolites into Milk of Dairy Cows. Food Addit Contam. Part A Chem. Anal. Control. Expo. Risk Assess..

[56] Dinleyici M., Aydemir O., Yildirim G.K., Kaya T.B., Carman K.B. (2018). Human Mature Milk Zearalenone and Deoxynivalenol Levels in Turkey. Neuro Endocrinol. Lett..

[57] Kolpin D.W., Schenzel J., Meyer M.T., Phillips P.J., Hubbard L.E., Scott T.M., Bucheli T.D. (2014). Mycotoxins: Diffuse and Point Source Contributions of Natural Contaminants of Emerging Concern to Streams. Sci. Total Environ..

[58] Schenzel J., Schwarzenbach R.P., Bucheli T.D. (2010). Multi-Residue Screening Method to Quantify Mycotoxins in Aqueous Environmental Samples. J. Agric. Food Chem..

[59] Ribeiro A.R., Maia A., Santos M., Tiritan M.E., Ribeiro C.M. (2016). Occurrence of Natural Contaminants of Emerging Concern in the Douro River Estuary, Portugal. Arch. Environ. Contam. Toxicol..

[60] Dombrink-Kurtzman M.A., Poling S.M., Kendra D.F. (2010). Determination of Deoxynivalenol in Infant Cereal by Immunoaffinity Column Cleanup and High-Pressure Liquid Chromatography-UV Detection. J. Food Prot..

[61] Bucheli T.D., Wettstein F.E., Hartmann N., Erbs M., Vogelgsang S., Forrer H.R., Schwarzenbach R.P. (2008). *Fusarium* Mycotoxins: Overlooked Aquatic Micropollutants?. J. Agric. Food Chem..

[62] Do K.H., An T.J., Oh S.K., Moon Y. (2015). Nation-Based Occurrence and Endogenous Biological Reduction of Mycotoxins in Medicinal Herbs and Spices. Toxins.

[63] Dong F., Xing Y.J., Lee Y.W., Mokoena M.P., Olaniran A.O., Xu J.H., Shi J.R. (2020). Occurrence of *Fusarium* Mycotoxins and Toxigenic *Fusarium* Species in Freshly Harvested Rice in Jiangsu, China. World Mycotoxin J..

[64] Delgado J.A., Schwarz P.B., Gillespie J., Rivera-Varas V.V., Secor G.A. (2010). Trichothecene Mycotoxins Associated with Potato Dry Rot Caused by *Fusarium* Graminearum. Phytopathology.

[65] Pal S., Singh N., Ansari K.M. (2017). Toxicological Effects of Patulin Mycotoxin on the Mammalian System: An Overview. Toxicol. Res. (Camb).

[66] Graziani F., Pinton P., Olleik H., Pujol A., Nicoletti C., Sicre M., Quinson N., Ajandouz E., Perrier J., Pasquale E. (2019). Deoxynivalenol Inhibits the Expression of Trefoil Factors (TFF) by Intestinal Human and Porcine Goblet Cells. Arch. Toxicol..

[67] Maresca M. (2013). From the Gut to the Brain: Journey and Pathophysiological Effects of the Food-Associated Trichothecene Mycotoxin Deoxynivalenol. Toxins.

[68] Pinton P., Graziani F., Pujol A., Nicoletti C., Paris O., Ernouf P., Di Pasquale E., Perrier J., Oswald I.P., Maresca M. (2015). Deoxynivalenol Inhibits the Expression by Goblet Cells of Intestinal Mucins through a PKR and MAP Kinase Dependent Repression of the Resistin-Like Molecule Beta. Mol. Nutr. Food Res..

[69] Roberts S.E., Thorne K., Thapar N., Broekaert I., Benninga M.A., Dolinsek J., Mas E., Miele E., Orel R., Pienar C. (2020). A Systematic Review and Meta-Analysis of Paediatric Inflammatory Bowel Disease Incidence and Prevalence Across Europe. J. Crohns Colitis.

[70] De Walle J.V., Sergent T., Piront N., Toussaint O., Schneider Y.J., Larondelle Y. (2010). Deoxynivalenol Affects in Vitro Intestinal Epithelial Cell Barrier Integrity through Inhibition of Protein Synthesis. Toxicol. Appl. Pharmacol..

[71] Pestka J.J. (2010). Deoxynivalenol-Induced Proinflammatory Gene Expression: Mechanisms and Pathological Sequelae. Toxins.

[72] Akbari P., Braber S., Gremmels H., Koelink P.J., Verheijden K.A.T., Garssen J., Fink-Gremmels J. (2014). Deoxynivalenol: A Trigger for Intestinal Integrity Breakdown. FASEB J..

[73] Vandenbroucke V., Croubels S., Verbrugghe E., Boyen F., De Backer P., Ducatelle R., Rychlik I., Haesebrouck F., Pasmans F. (2009). The Mycotoxin Deoxynivalenol Promotes Uptake of Salmonella Typhimurium in Porcine Macrophages, Associated with ERK1/2 Induced Cytoskeleton Reorganization. Vet. Res..

[74] Vandenbroucke V., Croubels S., Martel A., Verbrugghe E., Goossens J., Van Deun K., Boyen F., Thompson A., Shearer N., De Backer P. (2011). The Mycotoxin Deoxynivalenol Potentiates Intestinal Inflammation by Salmonella Typhimurium in Porcine Ileal Loops. PLoS ONE.

[75] Chung Y., Zhou H., Pestka J.J. (2003). Transcriptional and Posttranscriptional Roles for p38 Mitogen-Activated Protein Kinase in Upregulation of TNF-Alpha Expression by Deoxynivalenol (Vomitoxin). Toxicol. Appl. Pharmacol..

[76] Adesso S., Autore G., Quaroni A., Popolo A., Severino L., Marzocco S. (2017). The Food Contaminants Nivalenol and Deoxynivalenol Induce Inflammation in Intestinal Epithelial Cells by Regulating Reactive Oxygen Species Release. Nutrients.

[77] Gan F., Lin Z., Tang J., Chen X., Huang K. (2023). Deoxynivalenol at no-Observed Adverse-Effect Levels Aggravates DSS-Induced Colitis through the JAK2/STAT3 Signaling Pathway in Mice. J. Agric. Food Chem..

[78] Strati F., Cavalieri D., Albanese D., De Felice C., Donati C., Hayek J., Jousson O., Leoncini S., Renzi D., Calabro A. (2017). New Evidences on the Altered Gut Microbiota in Autism Spectrum Disorders. Microbiome.

[79] Grenier B., Applegate T.J. (2013). Modulation of Intestinal Functions Following Mycotoxin Ingestion: Meta-Analysis of Published Experiments in Animals. Toxins.

[80] Kos J., Radic B., Lesic T., Anic M., Jovanov P., Saric B., Pleadin J. (2024). Climate Change and Mycotoxins Trends in Serbia and Croatia: A 15-Year Review. Foods.

[81] Furlong E.B., Buffon J.G., Cerqueira M.B., Kupski L. (2024). Mitigation of Mycotoxins in Food-is it Possible?. Foods.

[82] Sydenham S., De Villiers C. Fusarium Head Blight Incidence Is on the Rise, Globally. https://www.grainsa.co.za/fusarium-head-blight-incidence-is-on-the-rise,-globally.

[83] Zhao Y., Selvaraj J.N., Xing F., Zhou L., Wang Y., Song H., Tan X., Sun L., Sangare L., Folly Y.M. (2014). Antagonistic Action of Bacillus Subtilis Strain SG6 on *Fusarium graminearum*. PLoS ONE.

[84] Czaczyk K., Trojanowska K., Mueller A. (2002). Antifungal Activity of Bacillus Coagulans against *Fusarium* Sp.. Acta Microbiol. Pol..

[85] Garcia G.R., Payros D., Pinton P., Dogi C.A., Laffitte J., Neves M., Gonzalez Pereyra M.L., Cavaglieri L.R., Oswald I.P. (2018). Intestinal Toxicity of Deoxynivalenol is Limited by Lactobacillus Rhamnosus RC007 in Pig Jejunum Explants. Arch. Toxicol..

[86] McCormick S.P. (2013). Microbial Detoxification of Mycotoxins. J. Chem. Ecol..

[87] Rabbee M.F., Ali M.S., Choi J., Hwang B.S., Jeong S.C., Baek K.H. (2019). Bacillus Velezensis: A Valuable Member of Bioactive Molecules within Plant Microbiomes. Molecules.

[88] Li F., Wang J., Huang L., Chen H., Wang C. (2017). Effects of Adding *Clostridium* Sp. WJ06 on Intestinal Morphology and Microbial Diversity of Growing Pigs Fed with Natural Deoxynivalenol Contaminated Wheat. Toxins.

[89] United States Department of Agriculture Grain Fungal Diseases and Mycotoxin Reference. 2006, *STOP 3630*, 1. https://www.ams.usda.gov/sites/default/files/media/FungalDiseaseandMycotoxinReference2017.pdf.

[90] Karlovsky P., Suman M., Berthiller F., De Meester J., Eisenbrand G., Perrin I., Oswald I.P., Speijers G., Chiodini A., Recker T. (2016). Impact of Food Processing and Detoxification Treatments on Mycotoxin Contamination. Mycotoxin Res..

[91] Zhang K., Wong J.W., Jia Z., Vaclavikova M., Trucksess M.W., Begley T.H. (2014). Screening Multimycotoxins in Food-Grade Gums by Stable Isotope Dilution and Liquid Chromatography/Tandem Mass Spectrometry. J. AOAC Int..

[92] Keller N.P., Turner G., Bennett J.W. (2005). Fungal Secondary Metabolism - from Biochemistry to Genomics. Nat. Rev. Microbiol..

[93] Turner P.C., Taylor E.F., White K.L., Cade J.E., Wild C.P. (2009). A Comparison of 24 H Urinary Deoxynivalenol with Recent V. Average Cereal Consumption for UK Adults. Br. J. Nutr..

[94] Turner P.C., Hopton R.P., Lecluse Y., White K.L., Fisher J., Lebailly P. (2010). Determinants of Urinary Deoxynivalenol and De-Epoxy Deoxynivalenol in Male Farmers from Normandy, France. J. Agric. Food Chem..

[95] Chin V.K., Yong V.C., Chong P.P., Amin Nordin S., Basir R., Abdullah M. (2020). Mycobiome in the Gut: A Multiperspective Review. Mediators Inflamm..

[96] Suhr M.J., Hallen-Adams H.E. (2015). The Human Gut Mycobiome: Pitfalls and Potentials—A Mycologist’s Perspective. Mycologia.

[97] Nalage D., Sontakke T., Biradar A., Jogdand V., Kale R., Harke S., Dixit P. (2023). The Impact of Environmental Toxins on the Animal Gut Microbiome and their Potential to Contribute to Disease. Food Chem. Adv..

[98] Maresca M., Yahi N., Younes-Sakr L., Boyron M., Caporiccio B., Fantini J. (2008). Both Direct and Indirect Effects Account for the Pro-Inflammatory Activity of Enteropathogenic Mycotoxins on the Human Intestinal Epithelium: Stimulation of Interleukin-8 Secretion, Potentiation of Interleukin-1beta Effect and Increase in the Transepithelial Passage of Commensal Bacteria. Toxicol. Appl. Pharmacol..

[99] Graziani F., Pujol A., Nicoletti C., Pinton P., Armand L., Di Pasquale E., Oswald I.P., Perrier J., Maresca M. (2015). The Food-Associated Ribotoxin Deoxynivalenol Modulates Inducible NO Synthase in Human Intestinal Cell Model. Toxicol. Sci..

[100] Venkatesh N., Keller N.P. (2019). Mycotoxins in Conversation with Bacteria and Fungi. Front. Microbiol..

[101] Etzel R.A. (2014). Reducing Malnutrition: Time to Consider Potential Links between Stunting and Mycotoxin Exposure?. Pediatrics.

[102] Sa S.V.D., Faria M.A., Fernandes J.O., Cunha S.C. (2024). In Vitro Digestion and Intestinal Absorption of Mycotoxins due to Exposure from Breakfast Cereals: Implications for Children’s Health. Toxins.

[103] Coates A.E., Hardman C.A., Halford J.C.G., Christiansen P., Boyland E.J. (2019). Social Media Influencer Marketing and Children’s Food Intake: A Randomized Trial. Pediatrics.

[104] Lappe A. (2013). Marketing Food to Children.

[105] Mager D.R., Marcon M., Brill H., Liu A., Radmanovich K., Mileski H., Nasser R., Alzaben A., Carroll M.W., Yap J. (2018). Adherence to the Gluten-Free Diet and Health-Related Quality of Life in an Ethnically Diverse Pediatric Population with Celiac Disease. J. Pediatr. Gastroenterol. Nutr..

[106] Wolf R.L., Lebwohl B., Lee A.R., Zybert P., Reilly N.R., Cadenhead J., Amengual C., Green P.H.R. (2018). Hypervigilance to a Gluten-Free Diet and Decreased Quality of Life in Teenagers and Adults with Celiac Disease. Dig. Dis. Sci..

[107] Kresse M., Drinda H., Romanotto A., Speer K. (2019). Simultaneous Determination of Pesticides, Mycotoxins, and Metabolites as Well as Other Contaminants in Cereals by LC-LC-MS/MS. J. Chromatogr. B Analyt Technol. Biomed. Life. Sci..

